# Decreased expression of FcγRII in active Graves' disease patients

**DOI:** 10.1002/jcla.22904

**Published:** 2019-04-29

**Authors:** Xixuan Lu, Shiqiao Peng, Xinyi Wang, Zhongyan Shan, Weiping Teng

**Affiliations:** ^1^ Department of Endocrinology and Metabolism, Institute of Endocrinology, Liaoning Provincial Key Laboratory of Endocrine Diseases The First Affiliated Hospital of China Medical University Shenyang China; ^2^ Department of Laboratory Medicine The First Affiliated Hospital of China Medical University Shenyang China

**Keywords:** autoimmunity, CD32B, FcγR, FcγRII, Graves' disease

## Abstract

**Background:**

Graves' disease (GD) is a common autoimmune disease characterized by genetic and environmental factors. Fcγ receptors (FcγRs) are involved in several autoimmune disorders through recognizing immunoglobulin (Ig) G antibodies and mediating immune response. The study on the expression of FcγRs in GD patients is scarce. The purpose of this study was to evaluate the expression of three different types of FcγRs in patients with active and remissive GD.

**Methods:**

Blood samples of patients and healthy subjects were collected to analyze the percentage of FcγRI (CD64), FcγRII (CD32), and FcγRIII (CD16) on peripheral blood mononuclear cells (PBMCs) and monocytes by flow cytometry and Western blotting. CD32 isotypes were also examined in cases and controls by real‐time PCR.

**Results:**

The cell percentages expressed CD32 and protein expressions of CD32 on PBMCs, and monocytes from patients with active GD were significantly reduced compared to controls and patients with remissive GD. In particular, the expression of CD32B on PBMC was also decreased in active GD patients. However, the cell percentages expressed CD16 and CD64 from PBMCs and monocytes were comparable between three groups. Besides, the percentages of CD14^+^CD32^+^ cells were negatively correlated with TRAb titers in active GD patients (*r* = −0.5825, *P *＜ 0.001).

**Conclusion:**

These results suggested that CD32 may act as a novel marker for active GDs. The expression of monocytic CD32, in particular CD32B, in GD patients might play a crucial role in maintaining FcγRs function and be a therapeutic target in GD patients.

## 1 INTRODUCTION

Graves' disease is a common autoimmune disorder that has a multi‐faced etiology involving genetic and environmental factors and is featured by the presence of thyroid stimulating hormone receptor (TSHR)‐stimulating antibodies (TSAbs) that can promote the function of thyroid follicular cells (TFCs) and stimulate thyroid growth, and thus lead to an overproduction of thyroid hormones (TH).^1^ It is reported that worldwide prevalence of Graves' disease (GD) has become popular with an incidence of 1 in 4000 persons per year.^2^ GD has generally been viewed to be mediated by a humoral autoimmune response mainly because the infiltration of lymphocytes can activate the TSHR‐reactive B cells and lead to the abundant production of TSAbs, manifesting as hyperthyroidism.^3^

Anti‐TSHR antibodies, as the diagnostic and therapeutic hallmarks of GD, can be found in 90% of GD patients and mainly belong to IgG1 class involved in the early period of humoral response.^4^ IgG exerts the immunoregulatory effects through FcγRs, a well‐acknowledged class of cell surface receptors that interact with the Fc domain of IgG. FcγRs are classed into activatory or inhibitory form according to different intracellular signaling motifs. There are three different FcγR classes in human beings including FcγRI (CD64), FcγRII (CD32), and FcγRIII (CD16). FcγRIIB is the only known inhibitory FcγR while others are activatory. FcγRs are broadly expressed in almost all hematopoietic cells except T cells. Most cells express activating as well as inhibitory FcγRs with the exception of NK cells (only expressing FcγRIII) and B cells (only expressing FcγRIIB).^5^ Activation of activatory FcγRs causes immune responses such as antibody‐dependent cell‐mediated cytotoxicity (ADCC) and antibody‐dependent cell‐mediated phagocytosis (ADCP), while crosslinking of the inhibitory FcγRIIB such as colligation of Fc*γ*RIIB with BCR can negatively regulate the activation and proliferation of B cell and therefore inhibit IgG production.^6^ These three types of Fc*γ*Rs are all expressed on human monocytes. CD64 is a highly affinitive receptor presented at considerable expression by monocytes.^7^ CD32 can also be detected at substantial levels on monocytes, with low affinity of two different isotypes. CD16 is a receptor with average affinity for IgG, which is expressed on approximately 10% of monocytes.^8^ Of these Fc*γ*Rs, CD16, CD32A, and CD64 are activatory Fc*γ* receptors with intracytoplasmic tyrosine‐based activatory motifs that induce monocytes initiation under receptor polymerization. In contrast, CD32B is composed of immunoreceptor tyrosine‐based inhibitory motif and serves as the only inhibitory Fc*γ*R via interacting with immune complexes.^9^

Autoantibodies are the key serological markers for many autoimmune diseases and also act as potential initiator of inflammation and contribute to disease development. FcγRs distinctly impact antigen intake and processing, cellular activating, and secretion of inflammatory regulators. There have been several studies indicating an involvement of FcγRs in autoimmune diseases like rheumatoid arthritis (RA),^10^, ^11^ systemic lupus erythematosus (SLE),^12^, ^13^ and immune thrombocytopenia (ITP).^14^ Studies on the expression of FcγRs in autoimmune thyroid diseases are scarce. A study about Hashimoto's thyroiditis recently implied that increasing expression of FcγRIII and decreasing expression of FcγRIIB may be involved in the pathogenesis of HT.^15^ The expression of FcγRII from GD patients was reported by Estienne et al, and the authors suggested that FcγRII expressed by thyrocytes might involve in eliminating IgG.^16^

Here, we aimed to investigate the potential role of FcγRs in active and remissive GD patients. The expression of FcγRI (CD64), FcγRII (CD32), and FcγRIII (CD16) on peripheral blood mononuclear cells (PBMCs) and monocytes from GD patients were examined by flow cytometry and Western blotting. The CD32 isotypes (CD32A and CD32B) were studied on PBMCs from cases and controls by real‐time PCR as well. Moreover, we also analyzed the correlation between the expression levels of FcγRs with autoantibodies.

## 2 METHODS

### 2.1 Subjects and study group

A total of 76 subjects were enrolled in this study, including 30 patients with GD in active, 16 patients with GD in remission, and 30 age‐ and sex‐matched controls. Patients with active GD were newly diagnosed with no medication before by a standard clinical and laboratory examination, including the history and physical examination, serological detection, and radioactive iodine uptake assay. GD patients in remission had been treated with methimazole for over 3 months, and the clinical characteristic had been dramatically improved compared with those in active patients. All participants had no history of systemic autoimmune disease, chronic disease, or any severe illnesses. The detection of serum thyrotropin (TSH), free T3 (FT3), free T4 (FT4), and thyroid autoantibodies including TPOAb, TgAb, and thyrotrophin receptor antibody (TRAb) was performed using electrochemiluminescent immunoassay with Cobas Elesys 601 (Roche Diagnostics Ltd, Switzerland). All information was listed in Table 1.

**Table 1:** The clinical characteristics of patients with Graves' disease and healthy controls

Variable	HC	GD in active	GD in remission	Normal range
No.	30	30	16	—
Age (y)	37 ± 8	30 ± 9	36 ± 5	—
Gender (M/F)	4/26	5/25	3/13	—
TSH (mIU/L)	1.96 ± 0.27	0.007 ± 0.01	2.34 ± 0.35	0.35‐4.94
FT4 (pmol/L)	13.4 ± 1.54	46.02 ± 14.7	15.79 ± 2.15	9.01‐19.05
FT3 (pmol/L)	3.18 ± 0.55	34.3 ± 6.90	3.96 ± 0.71	2.63‐5.70
TRAb (IU/L)	—	27.9 ± 9.52	2.3 ± 0.43	0‐1.75
TPOAb (IU/mL)	5.03 ± 0.17	167.5 ± 23.23	24.3 ± 11.62	0.11‐5.23
TgAb (IU/mL)	2.54 ± 0.36	192.28 ± 17.32	47.12 ± 15.97	0.81‐3.83

Note

Data are expressed as means ± standard deviation. M, male; F, female. “—” represents that the experiment was not performed, or the data are not applicable.

Abbreviation(s): GD, Graves' disease; HC, healthy controls.

### 2.2 Peripheral blood mononuclear cell isolation

Fresh blood was collected in heparinized tubes. The isolation of PBMC was performed by Ficoll‐isopaque density gradient centrifugation (Gibco BRL, Life Technologies Ltd) as described previously.^17^ About 1 ml Trizol (Invitrogen) was added into cells and stored in −80°C for further use.

### 2.3 Western blot analysis

Peripheral blood mononuclear cells were homogenized in ice PBS containing 0.05% Triton X‐100 and protease inhibitor cocktail (Sigma). Protein samples were run on 10% sodium dodecyl sulfate‐polyacrylamide gel electrophoresis (Sigma) and transferred onto PVDF membranes according to standard protocol, and then blocked with 5% dried skimmed milk in TBST for at least 1h. The membranes were then incubated overnight at 4°C, either with a mouse monoclonal anti‐CD64 antibody and anti‐CD32 antibody (1:800; Abcam), a polyclonal rabbit anti‐CD16 antibody (1:800; Abcam), or a polyclonal rabbit anti‐GAPDH (1:1000; Abcam), and then incubated with horseradish peroxidase‐conjugated secondary antibodies (1:1000; Bio‐Rad) at room temperature for 1 hour after washing three times using TBST. Blots were washed again and developed using an enhanced chemiluminescence kit (Amersham Pharmacia Biotech). Immunoblot bands quantification was calculated by means of a Bio‐Rad calibrated densitometer (GS‐800) using the vendor's software (Bio‐Rad Laboratories); GAPDH was used as an internal reference for analyses.

### 2.4 Flow cytometric analysis of the proportion of CD64/CD32/CD16 cells on PBMCS and monocytes

Mononuclear cells from the PBMCs were harvested and subsequently stimulated with PMA (Sigma‐Aldrich), ionomycin (Sigma‐Aldrich), and monensin (BD Biosciences Corp) for 8 hours in a thermostat. The cells were stained with a CD14‐FITC monoclonal antibody (eBioscience), and the surface expression of FcγRs on monocytes was investigated by incubating with monoclonal antibodies against CD16‐APC (BD Biosciences Corp.), CD32‐APC (DAKO), and CD64‐APC (BD Biosciences Corp.) for 30 minutes in darkness. Cells were then washed with PBS for three times and fixed in 1% paraformaldehyde. The analysis of samples was performed using a BD FACS Array (Becton‐Dickinson) equipped with Cellquest software. Gates for monocytes were set according to forward scatter pattern.

### 2.5 Quantitative real‐time PCR analysis

Total RNA was extracted from PBMC using TRIZOl (Invitrogen). Reverse transcription was achieved using Takara Kit (RR047A). cDNA amplification was then performed using SYBR Premix Ex Taq (TAKARA, RR820) in Roche 480 Light Cycler. PCR was carried out for 40 cycles according to the following procedure: at 95°C for 30 s, annealing for 30 s, and at 60°C for 34 s. Each sample was analyzed in duplicate. Relative mRNA expression levels of the target genes were calculated using GAPDH as an internal control. The primer sequences used for PCR are shown as Table 2.

**Table 2:** Sequences of primers used to amplify

Genes	Forward primer	Reverse primer
CD32B	AGCCAATCCCACTAATCCTGA	GGTGCATGAGAAGTGAATAGGTG
CD32A	CTAAGCTTGTCTCTTAAAACCCAC	CAGCAGCAAAACTGTCAATGGTT
GAPDH	CAA AGACCTGTACGCCAACA	CAA AGACCTGTACGCCAACA

### 2.6 Statistical analysis

All statistical analyses were performed using SPSS Software 20.0 (SPSS, Inc). Results are given as means ± standard deviation. One‐way analysis of variance (ANOVA) was used for the comparison of the expression of FcγRs in all three groups. Spearman's method was used for the correlation analysis between FcγRs and clinical characteristic. GraphPad Prism 5 software was used to analyze data and create graphs. *P* value < 0.05 was viewed as statistically significant.

## 3 RESULTS

### 3.1 Clinical characteristics of Grave' disease patients in active and in remission and controls

The clinical features and laboratory parameters are presented in Table 1. We enrolled 46 patients (30 initial cases, 16 remissive cases) and 30 age‐ and gender‐matched healthy controls. As shown in Table 1, patients in active group exhibited significantly higher levels of FT3, FT4, TPOAb, TgAb, and TRAb and extremely lower levels of TSH than controls. In addition, patients in remissive group remained euthyroid with greatly improved laboratory parameters as compared with patients in active group.

### 3.2 Protein expression of FCγRI (CD64), FCγRII (CD32), and FCγRIII (CD16) on peripheral blood mononuclear cells from Grave' disease in active and in remission and controls

**Figure 1:**
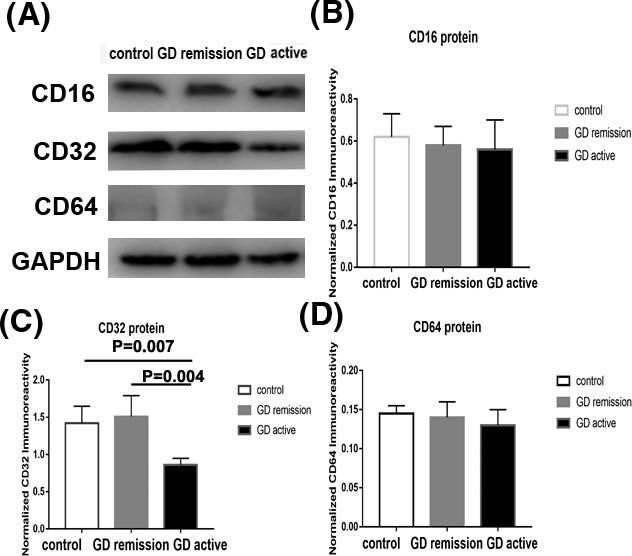
Protein expression of FcγRI (CD64), FcγRII (CD32), and FcγRIII (CD16) on peripheral blood mononuclear cells (PBMCs) from Grave' disease (GD) in active and in remission and controls by Western blotting. A, Western blot analysis of CD16, CD32, and CD64 expression in PBMCs from GD in active and in remission and controls. B, The ratio of CD16/GAPDH was determined to give a mean net density. C, The ratio of CD32/GAPDH was determined to give a mean net density. D, The ratio of CD64/GAPDH was determined to give a mean net density. Data are presented as the means ± standard error. One‐way ANOVA was used to compare the differences in multiple groups. *P < 0.05* was considered significant. GD, Graves' disease

The expression of FcγRs on PBMCs from GD patients and controls was studied by Western blotting. As shown in Figure 1A,B, CD16 expression on PBMCs remained no difference between three groups (*P >* 0.05, respectively). Moreover, the results also shown that CD64 expression on PBMCs was comparable among GD patients (both initial and remissive) and controls (*P *> 0.05, respectively; Figure 1A,D). However, as the data presented in Figure 1A,C, GD patients in active revealed a lower expression of CD32 on PBMCs compared to those with GD in remission and controls (*P* < 0.05, respectively).

### 3.3 The surface expression of CD64, CD32, AND CD16 on PBMCS from GD cases and controls

**Figure 2:**
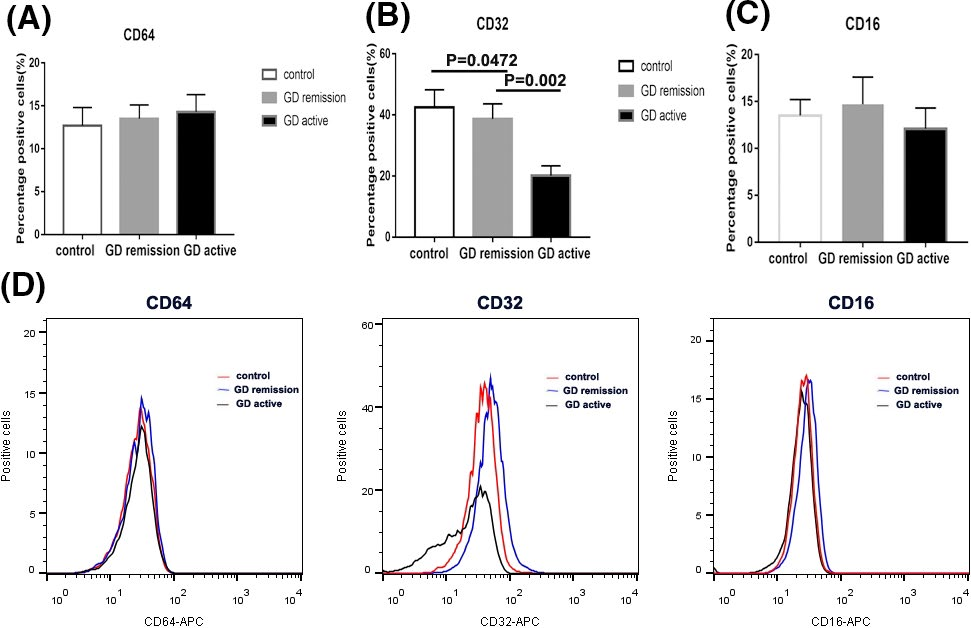
The percentage of FcγRI (CD64), FcγRII (CD32), and FcγRIII (CD16) on peripheral blood mononuclear cells (PBMCs) from Grave' disease (GD) in active and in remission and controls by flow cytometric analysis. A, Bar graphs from three groups depicting cell percentage of CD64‐enriched PBMCs (left panels). B, Bar graphs from three groups depicting cell percentage of CD32‐enriched PBMCs (middle panels). C, Bar graphs from three groups depicting cell percentage of CD16‐enriched PBMCs (right panels). D, Flow cytometry histogram from three groups depicting cell percentage of FcγR‐enriched PBMCs. Data are presented as the means ± standard error. One‐way ANOVA was used to compare the differences in multiple groups. *P < 0.05* was considered significant. GD, Graves' disease

We examined the surface expression of three FcγRs on PBMCs from patients and healthy subjects by flow cytometry study. As demonstrated in Figure 2A,D, there is no significant difference in the cell percentages of CD64 from patients with GD and controls (14.32 ± 2.03 for GD in active vs. 13.52 ± 1.61 for GD in remission vs. 12.74 ± 2.12 for controls; *P *> 0.05). In addition, we also could not find difference in the proportion of CD16 among three groups (12.12 ± 2.21 for GD in active vs. 13.74 ± 2.92 for GD in remission vs. 13.54 ± 1.75 for controls; *P *> 0.05; Figure 2C,D). However, as demonstrated in Figure 2B,D, the cell percentages of CD32 were remarkably lower in initial GD group (20.13 ± 3.21) as compared with those in remission (38.72 ± 4.96; *P *< 0.05) and controls (42.5 ± 5.73; *P *< 0.05).

### 3.4 The expression of CD64, CD32, AND CD16 on monocytes from GD patients and controls

**Figure 3:**
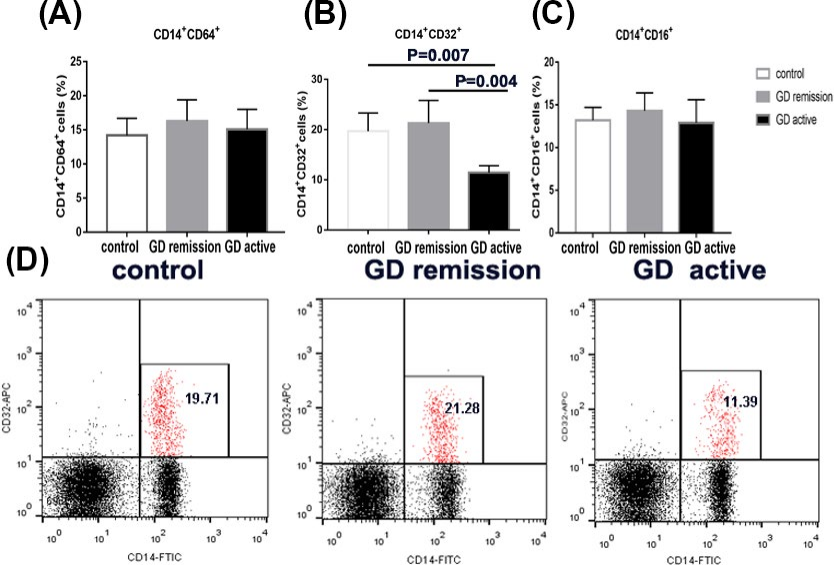
The percentage of FcγRI (CD64), FcγRII (CD32), and FcγRIII (CD16) on monocytes from Grave' disease (GD) in active and in remission and controls by flow cytometric analysis. A, Bar graphs from three groups depicting cell percentage of CD64‐enriched monocytes (left panels). B, Bar graphs from three groups depicting cell percentage of CD32‐enriched monocytes (middle panels). C, Bar graphs from three groups depicting cell percentage of CD16‐enriched monocytes (right panels). D, Flow cytometry dot plots from three groups depicting CD32 expression by CD14‐enriched monocyte cells. The number in the right quadrants represents the percentage of CD14^+^CD32^+^ in each group. Data are presented as the means ± standard error. One‐way ANOVA was used to compare the differences in multiple groups. *P < 0.05* was considered significant. GD, Graves' disease

**Figure 4:**
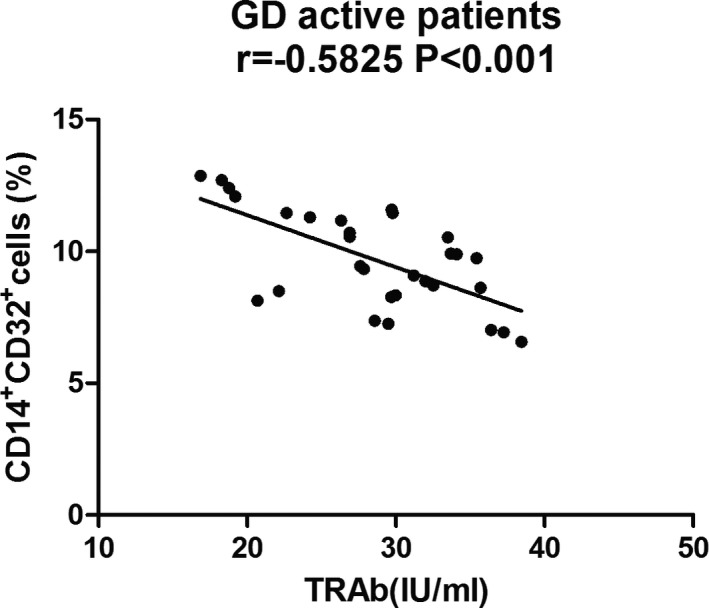
Correlation analysis of laboratory parameters and cell percentage of CD14^+^CD32^+^cells in active GD patients. Association between CD32 expression by CD14‐enriched monocyte cells and TRAb in GD patients. The correlation between CD14^+^CD32^+^ and clinical characteristic was analyzed with Spearman's method. TRAb, TSH receptor antibody; GD, Graves' disease

The percentages of CD14^+^ CD64^+^cells, CD14^+^ CD32^+^ cells, and CD14^+^ CD16^+^ cells were measured in the PBMCs from GD patients in active and in remission and healthy control subjects. As shown in Figure 4A, the cell percentages expressed CD64 in monocytes were comparable between GD patients and control subjects (15.16 ± 2.92 for GD in active vs. 16.37 ± 3.1 for GD in remission vs 14.25 ± 2.5 for controls; *P *> 0.05). Moreover, no differences were found in the cell percentages expressed CD16 between patient group and control group (12.91 ± 2.73 for GD in active vs. 14.3 ± 2.11 for GD in remission vs. 13.24 ± 1.55 for controls; *P *> 0.05; Figure 3C). However, as shown in Figure 3B, the cell proportions expressed CD32 in monocytes were significantly reduced in initial GD patients (11.39 ± 1.41) as compared with patients in remission (21.28 ± 4.53; *P* < 0.05) and healthy subjects (19.71 ± 3.59; *P* < 0.05). There was no difference between patients in remission and healthy controls.

### 3.5 Association between laboratory parameters and cell percentage of CD14^+^CD32^+^cells in active GD patients

In this study, we also analyzed the association of clinical and laboratory characteristic with the expression of FcγRs. As exhibited in Figure 4, an inverse correlation between the cell percentage of CD14^+^CD32^+^ cells and TRAb titers was found in the active patients (*r* = −0.5825, *P *< 0.001). No significant relationship was found between laboratory factors and the expression of other FcγRs (*P *> 0.05).

### 3.6 CD32B mRNA expression in PBMCS from GD patients in active and in remission and control subjects

**Figure 5:**
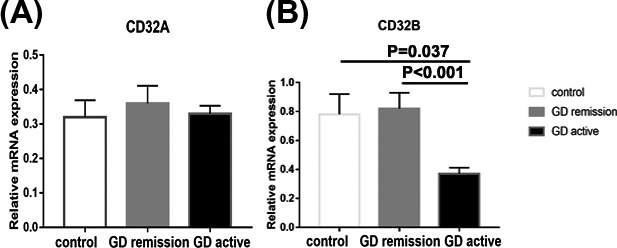
Bar graphs comparing CD32A and CD32B mRNA expression peripheral blood mononuclear cells (PBMCs) from Grave' disease (GD) in active and in remission and controls by real‐time PCR. CD32A (A) and CD32B (B) expression was examined by real‐time PCR using GAPDH as an endogenous reference. The results are shown as fold changes of CD32A and CD32B gene expression in GD patients in active and remission compared with the controls. Data are presented as the means ± standard error. One‐way ANOVA was used to compare the differences in multiple groups. *P < 0.05* was considered significant. GD, Graves' disease

To further determine the role of CD32 subtypes, we analyzed CD32 isotypes in PBMCs from GD patients and controls. As shown in Figure 5A, there was no difference in the mRNA expression of CD32A between GD patients and controls (*P *> 0.05). However, the mRNA expression of CD32B was significantly lower in patients with initial GD than those with remissive GD and healthy subjects (*P *< 0.05).

## 4 DISCUSSION

The pathogenesis of GD is involved by cellular and humoral factors, and the latter is featured by the presence of TRAbs. The involvement of autoantibodies to promote autoimmunity greatly depends on their ability to engage with Fcγ receptors which are expressed by immune cells. FcγRs are contributed to binding immune complexes and transmitting signaling and subsequently affect cellular functions.^18^ The interplay between pathogen‐dependent IgG and the activation of FcγRs regulates pathogen elimination via ADCC and the reduction of cytotoxic cells and exerts a crucial role in secreting cytokines and inflammatory regulators.^6^ FcγRIIB can inhibit all the processes and therefore downregulates immune complex‐mediated cellular activation.^19^ Hence, the FcγRs are pivotal to the immune system via exerting different functions.

FcγRs were found to be involved in many autoimmune diseases including SLE, RA. So far, limited data are yet available regarding FcγRs expression from PBMCs and monocytes in GD. In this study, we reported for the first time that decreased cell percentages expressed FcγRII (CD32) as well as decreased protein levels of FcγRII from PBMCs and monocytes have been found in patients with active GD patients by using flow cytometry and Western blotting, and we further verified the isotype of CD32 was CD32B. Our result was consistent with the previous study, which found that the expression of CD32 was reduced in PBMC from HT patients,^15^ suggesting that the upregulated antibody‐dependent cellular cytotoxicity was activated in AITD. The pathology of GD is involved by both Th1‐ and Th2‐mediated immune activity. The reduction of CD32 in PBMCs and monocytes in this study may partly be explained by the influence of inflammatory cytokines since both Th1 cytokines (TNF‐α and IFN‐γ) and Th2 cytokines (IL‐4 and IL‐10) can affect the expression of FcγRs.^9^, ^20^ It was reported that CD32B expression was increased by IL‐4 and decreased by IFN‐γ while CD32A was decreased by IL‐4 and increased by IFN‐γ.^9^ Although GD was mainly viewed as a predominant Th2‐mediated immune response, the infiltration in the intrathyroidal of both Th1 and Th2 cells also suggested a role of Th1 cytokines. The shift of Th1‐Th2 balance may contribute to the aberrant expression of CD32. Besides, abnormal clearance of immune complexes and apoptotic fragment in GD might also involve in the expression of CD32.^21^ In summary, the imbalance between cellular immunity and the cytokines production in GD patients was involved in the reduction of CD32B in monocytes, which might ultimately aggravate the process of GD via several regulations to different cells. More researches are needed to illuminate this theory.

In our study, we found CD64 expression on PBMCs and monocytes were comparable between patients and controls. The expression of CD64 on monocytes has been examined in SLE patients and was found to be increased in active systemic inflammation and be related to the development of nephritis.^22^ These results were concordant with the study done by Liu et al in HT patients.^15^ In addition, we also found cell percentages expressed CD16 were also similar among three groups, which was inconsistent with those in HT study. This discrepancy may due to the lower patients' number and the different mechanism between HT and GD. Further studies are needed to clarify these results.

To date, this is the first report to study CD32 isotypes in PBMCs from GD patients. Our results showed that the expression of CD32B on PBMCs was reduced in active GD patients, which was consistent with other studies on CD32B expression in patients with RA and SLE.^13^, ^23^ Previous study also showed that the CD32B expression on B cells was reduced in HT and SLE patients.^15^, ^24^ CD32B can prevent antibody‐mediated autoimmunity through regulating different cellular function—by reducing the production of autoantibodies via effects on B cells, by limiting the autoantibody‐dependent inflammation via influencing macrophages and neutrophils, and by prohibiting immune‐complexed autoantigen presentation to autoreactive T cells.^25^ The over‐secretion of CD32B by B cells has been shown to reduce the incidence of SLE mouse model.^26^ It has been reported that CD32B was increased on dendritic cells in untreated RA subjects with a low disease activity.^27^ Studies focused on FcγRs expression of thyrocytes from AITD suggested CD32B and FcRn may participate in the autoantigen presenting.^16^, ^20^ Our study here demonstrated that the expression of inhibitory CD32B from PBMC was also downregulated in GD patients, providing further evidence to confirm CD32B could be involved in the pathogenesis of GD.

In the present study, we found the expression of CD32 expression on monocytes from active GD patients was negatively correlated with the TRAb titers, indicating that the expression of CD32 may be associated with disease activity. This correlation was consistent with Yuasa's study that FcγR IIB‐deficient mice have elevated collagen‐specific IgG titers and have shown severer disease activity in collagen‐induced arthritis.^28^ One of the unique characteristics of GD is the presence of TSHR antibodies owing to the breakdown of immunity tolerance. In patients with GD, TSHR‐reactive B cells survive deletion and potentially present thyroid autoantigen to T cells inducing proinflammatory cytokines.^29^ This correlation between the expression of CD32 on monocytes and TRAb titers supported that CD32 played a central role in antibody‐mediated autoimmune disease through antibody‐dependent mechanism.

## 5 CONCLUSION

In this study, we showed for the first time that GD patients in active had a reduced CD32 expression on monocytes. The effective CD32 isotype on GD patients was CD32B. In addition, the expression of CD32 was negatively correlated with TRAb titer in active GD patients. These results suggest that CD32 may act as a novel marker for activated GD. The monocytic CD32, in particular CD32B, in GD patients might play a crucial role in the pathogenesis of GD and could be a therapeutic target.

## ETHICAL APPROVAL

This study was approved by the Ethics Committee of the First Affiliated Hospital of China Medical University, and all participants signed informed consents before any performances. All procedures were in compliance with the ethical standards and with the 1964 Helsinki declaration and its later amendments.
